# Supplementary material for: Genome-wide association analyses of autoimmune hypothyroidism reveal autoimmune and thyroid-specific contributions and an inverse relationship with cancer risk
Source: Nat Genet. 2026 Feb 26;58(3):550–9. doi: 10.1038/s41588-026-02521-1 (PMC12987720; doi:10.1038/s41588-026-02521-1)
Supplement: Supplementary file 1 — Reporting Summary [file 41588_2026_2521_MOESM1_ESM.pdf]

Eukaryotic cell lines

Policy information about cell lines and Sex and Gender in Research

Cell line source(s)	Zap-70 deficient Jurkat cells, P116 (ATCC ATCC® CRL-2676).
Authentication	P116 cells were authenticated by confirmation of Zap-70 deficiency by flow cytometry with Zap-70 specific antibodies.
Mycoplasma contamination	P116 cells were confirmed to be mycoplasma negative.
Commonly misidentified lines (See ICLAC register)	Not applicable.

Plants

Seed stocks	*Report on the source of all seed stocks or other plant material used. If applicable, state the seed stock centre and catalogue number. If plant specimens were collected from the field, describe the collection location, date and sampling procedures.*
Novel plant genotypes	*Describe the methods by which all novel plant genotypes were produced. This includes those generated by transgenic approaches, gene editing, chemical/radiation-based mutagenesis and hybridization. For transgenic lines, describe the transformation method, the number of independent lines analyzed and the generation upon which experiments were performed. For gene-edited lines, describe the editor used, the endogenous sequence targeted for editing, the targeting guide RNA sequence (if applicable) and how the editor was applied.*
Authentication	*Describe any authentication procedures for each seed stock used or novel genotype generated. Describe any experiments used to assess the effect of a mutation and, where applicable, how potential secondary effects (e.g. second site T-DNA insertions, mosiacism, off-target gene editing) were examined.*

Flow Cytometry

Plots

Confirm that:

☒ The axis labels state the marker and fluorochrome used (e.g. CD4-FITC).

☒ The axis scales are clearly visible. Include numbers along axes only for bottom left plot of group (a 'group' is an analysis of identical markers).

☒ All plots are contour plots with outliers or pseudocolor plots.

☒ A numerical value for number of cells or percentage (with statistics) is provided.

Methodology

Sample preparation	Zap70-deficient Jurkat cells were obtained from ATCC. Cells were fixed and permeablized with Cytofix/Cytoperm buffer (BD Biosciences) and stained with the indicated antibodies.
Instrument	CytoFLEX LX (Beckman Coulter) flow cytometer.
Software	Cytometry analysis was performed with FlowJo software.
Cell population abundance	Cell frequencies in each indicated gate are reported as percentages of total live singlet cells. No sorting was performed.
Gating strategy	Live cells were selected based on FSC/SSC profiles. Singlets were selected based on FSC-H/FSC-A profiles. Quadrant plots were generated for the indicated antibody markers.

☒ Tick this box to confirm that a figure exemplifying the gating strategy is provided in the Supplementary Information.

Hematological Registry and Clinical Biobank decision 18th June 2021, Arctic biobank P0844: ARC_2021_1001.

Note that full information on the approval of the study protocol must also be provided in the manuscript.

Field-specific reporting

Please select the one below that is the best fit for your research. If you are not sure, read the appropriate sections before making your selection.

☒ Life sciences ☐ Behavioural & social sciences ☐ Ecological, evolutionary & environmental sciences

For a reference copy of the document with all sections, see nature.com/documents/nr-reporting-summary-flat.pdf

Life sciences study design

All studies must disclose on these points even when the disclosure is negative.

Sample size	Full sample size of FinnGen and UKBB were used, this sample was more than twice as large as any previous study.
Data exclusions	Data and analysis QC as previously published in the flagship paper - https://www.nature.com/articles/s41586-022-05473-8
Replication	Homogeneity of effects between FinnGen and UKBB was used to confirm validity. Further replication was performed during the review process with MVP and is presented in Supplementary Table 1.
Randomization	NA
Blinding	NA

Reporting for specific materials, systems and methods

We require information from authors about some types of materials, experimental systems and methods used in many studies. Here, indicate whether each material, system or method listed is relevant to your study. If you are not sure if a list item applies to your research, read the appropriate section before selecting a response.

Materials & experimental systems

n/a	Involved in the study	
☐	☒	Antibodies
☐	☒	Eukaryotic cell lines
☒	☐	Palaeontology and archaeology
☒	☐	Animals and other organisms
☒	☐	Clinical data
☒	☐	Dual use research of concern
☒	☐	Plants

Methods

n/a	Involved in the study	
☒	☐	ChIP-seq
☐	☒	Flow cytometry
☒	☐	MRI-based neuroimaging

Antibodies

Antibodies used

Phospho-SLP-76 (Tyr128) Monoclonal Antibody (HNDZ55), APC, eBioscience (Thermo Fisher 17-9037-42).

Alexa Fluor® 488 anti-ZAP70 Phospho (Tyr319)/Syk Phospho (Tyr352) Antibody (Biolegend 683711).

PerCP/Cyanine5.5 anti-ZAP70 Phospho (Tyr292) Antibody (Biolegend 693810).

PE-Cy™7 Mouse Anti-Human CD69 (BD 560712).

Ultra-LEAF™ Purified anti-human CD3 Antibody (Biolegend 317326).

Ultra-LEAF™ Purified anti-human CD28 Antibody (Biolegend 302934).

PE anti-human/mouse ZAP-70 Antibody (Biolegend 313403).

Validation

All antibodies were commercially sourced with quality control and validation provided by the manufacturer.

Data

Policy information about availability of data

All manuscripts must include a data availability statement. This statement should provide the following information, where applicable:

- Accession codes, unique identifiers, or web links for publicly available datasets
- A description of any restrictions on data availability
- For clinical datasets or third party data, please ensure that the statement adheres to our policy

Full GWAS summary statistics for all phenotypes described in the paper will be available for download from a public Google Cloud platform storage bucket upon publication.

Research involving human participants, their data, or biological material

Policy information about studies with human participants or human data. See also policy information about sex, gender (identity/presentation), and sexual orientation and race, ethnicity and racism.

Reporting on sex and gender	Sex is used to refer to biological sex and is included as a GWAS covariate.
Reporting on race, ethnicity, or other socially relevant groupings	NA, FinnGen is a study of Finnish-ancestry individuals.
Population characteristics	Population characteristics of the FinnGen study are described in the flagship paper - https://www.nature.com/articles/s41586-022-05473-8
Recruitment	FinnGen involves recruitment from hospital clinics, healthy blood donors, and population epidemiology studies. Recruitment details are further described in the flagship paper - https://www.nature.com/articles/s41586-022-05473-8.
Ethics oversight	The FinnGen study (https://www.finngen.fi/en) is a public-private partnership founded in 2017, including Finnish universities, biobanks, and hospital districts, as well as several pharmaceutical companies. The aim is to collect both National Health Records and genetic data from 500,000 Finns. The study participants include patients with acute and chronic diseases, healthy volunteers, and population collections. R12 consists of 520,210 individuals (55% females and 45% males). Study subjects in FinnGen provided informed consent for biobank research, based on the Finnish Biobank Act. Alternatively, separate research cohorts, collected prior the Finnish Biobank Act came into effect (in September 2013) and the start of FinnGen (August 2017), were collected based on study-specific consents and later transferred to the Finnish biobanks after approval by Fimea (Finnish Medicines Agency), the National Supervisory Authority for Welfare and Health. Recruitment protocols followed the biobank protocols approved by Fimea. The Coordinating Ethics Committee of the Hospital District of Helsinki and Uusimaa (HUS) statement number for the FinnGen study, under which this research is conducted, is Nr HUS/990/2017.<br>The FinnGen study is approved by Finnish Institute for Health and Welfare (permit numbers: THL/2031/6.02.00/2017, THL/1101/5.05.00/2017, THL/341/6.02.00/2018, THL/2222/6.02.00/2018, THL/283/6.02.00/2019, THL/1721/5.05.00/2019 and THL/1524/5.05.00/2020), Digital and population data service agency (permit numbers: VRK43431/2017-3, VRK/6909/2018-3, VRK/4415/2019-3), the Social Insurance Institution (permit numbers: KELA 58/522/2017, KELA 131/522/2018, KELA 70/522/2019, KELA 98/522/2019, KELA 134/522/2019, KELA 138/522/2019, KELA 2/522/2020, KELA 16/522/2020), Findata permit numbers THL/2364/14.02/2020, THL/4055/14.06.00/2020, THL/3433/14.06.00/2020, THL/4432/14.06/2020, THL/5189/14.06/2020, THL/5894/14.06.00/2020, THL/6619/14.06.00/2020, THL/209/14.06.00/2021, THL/688/14.06.00/2021, THL/1284/14.06.00/2021, THL/1965/14.06.00/2021, THL/5546/14.02.00/2020, THL/2658/14.06.00/2021, THL/4235/14.06.00/2021, Statistics Finland (permit numbers: TK-53-1041-17 and TK/143/07.03.00/2020 (earlier TK-53-90-20) TK/1735/07.03.00/2021, TK/3112/07.03.00/2021) and Finnish Registry for Kidney Diseases permission/extract from the meeting minutes on 4th July 2019.<br>The Biobank Access Decisions for FinnGen samples and data utilized in FinnGen Data Freeze 12 include: THL Biobank BB2017_55, BB2017_111, BB2018_19, BB_2018_34, BB_2018_67, BB2018_71, BB2019_7, BB2019_8, BB2019_26, BB2020_1, BB2021_65, Finnish Red Cross Blood Service Biobank 7.12.2017, Helsinki Biobank HUS/359/2017, HUS/248/2020, HUS/430/2021 §28, §29, HUS/150/2022 §12, §13, §14, §15, §16, §17, §18, §23, §58, §59, HUS/128/2023 §18, Auria Biobank AB17-5154 and amendment #1 (August 17 2020) and amendments BB_2021-0140, BB_2021-0156 (August 26 2021, Feb 2 2022), BB_2021-0169, BB_2021-0179, BB_2021-0161, AB20-5926 and amendment #1 (April 23 2020) and it´s modifications (Sep 22 2021), BB_2022-0262, BB_2022-0256, Biobank Borealis of Northern Finland_2017_1013, 2021_5010, 2021_5010 Amendment, 2021_5018, 2021_5018 Amendment, 2021_5015, 2021_5015 Amendment, 2021_5015 Amendment_2, 2021_5023, 2021_5023 Amendment, 2021_5023 Amendment_2, 2021_5017, 2021_5017 Amendment, 2022_6001, 2022_6001 Amendment, 2022_6006 Amendment, 2022_6006 Amendment, 2022_6006 Amendment_2, BB22-0067, 2022_0262, 2022_0262 Amendment, Biobank of Eastern Finland 1186/2018 and amendment 22§/2020, 53§/2021, 13§/2022, 14§/2022, 15§/2022, 27§/2022, 28§/2022, 29§/2022, 33§/2022, 35§/2022, 36§/2022, 37§/2022, 39§/2022, 7§/2023, 32§/2023, 33§/2023, 34§/2023, 35§/2023, 36§/2023, 37§/2023, 38§/2023, 39§/2023, 40§/2023, 41§/2023, Finnish Clinical Biobank Tampere MH0004 and amendments (21.02.2020 & 06.10.2020), BB2021-0140 8§/2021, 9§/2021, §9/2022, §10/2022, §12/2022, 13§/2022, §20/2022, §21/2022, §22/2022, §23/2022, 28§/2022, 29§/2022, 30§/2022, 31§/2022, 32§/2022, 38§/2022, 40§/2022, 42§/2022, 1§/2023, Central Finland Biobank 1-2017, BB_2021-0161, BB_2021-0169, BB_2021-0179, BB_2021-0170, BB_2022-0256, BB_2022-0262, BB22-0067, Decision allowing to continue data processing until 31st Aug 2024 for projects: BB_2021-0179, BB22-0067,BB_2022-0262, BB_2021-0170, BB_2021-0164, BB_2021-0161, and BB_2021-0169, and Terveystalo Biobank STB 2018001 and amendment 25th Aug 2020, Finnish

nature portfolio

Corresponding author(s): Mary Pat Reeve, mary.reeve@helsinki.fi

Last updated by author(s): Nov 8, 2025

Reporting Summary

Nature Portfolio wishes to improve the reproducibility of the work that we publish. This form provides structure for consistency and transparency in reporting. For further information on Nature Portfolio policies, see our Editorial Policies and the Editorial Policy Checklist.

Statistics

For all statistical analyses, confirm that the following items are present in the figure legend, table legend, main text, or Methods section.

n/a	Confirmed	
☐	☒	The exact sample size (*n*) for each experimental group/condition, given as a discrete number and unit of measurement
☒	☐	A statement on whether measurements were taken from distinct samples or whether the same sample was measured repeatedly
☐	☒	The statistical test(s) used AND whether they are one- or two-sided<br>*Only common tests should be described solely by name; describe more complex techniques in the Methods section.*
☐	☒	A description of all covariates tested
☒	☐	A description of any assumptions or corrections, such as tests of normality and adjustment for multiple comparisons
☒	☐	A full description of the statistical parameters including central tendency (e.g. means) or other basic estimates (e.g. regression coefficient) AND variation (e.g. standard deviation) or associated estimates of uncertainty (e.g. confidence intervals)
☐	☒	For null hypothesis testing, the test statistic (e.g. *F*, *t*, *r*) with confidence intervals, effect sizes, degrees of freedom and *P* value noted<br>*Give P values as exact values whenever suitable.*
☐	☒	For Bayesian analysis, information on the choice of priors and Markov chain Monte Carlo settings
☒	☐	For hierarchical and complex designs, identification of the appropriate level for tests and full reporting of outcomes
☐	☒	Estimates of effect sizes (e.g. Cohen's *d*, Pearson's *r*), indicating how they were calculated

Our web collection on statistics for biologists contains articles on many of the points above.

Software and code

Policy information about availability of computer code

Data collection	NA
Data analysis	Central data analysis and processing pipelines used are freely available: fine-mapping pipeline (https://github.com/FINNGEN/finemapping-pipeline); meta-analysis (https://github.com/FINNGEN/META_ANALYSIS); genetic ancestry and PCA pipeline (https://github.com/FINNGEN/pca_kinship); and GWAS SAIGE pipeline (https://github.com/FINNGEN/saige-pipelines). Please see https://finngen.gitbook.io/documentation/ for a detailed description of data production and analysis including code used to run analyses. Please see https://github.com/FINNGEN/ for further code repositories used to run analyses in FinnGen. GWAS analysis was performed using REGENIE 2.2.4 using a logistic mixed model adjusted for age, sex, genotyping batch, and the first ten principal components of ancestry with an approximate Firth test for robust effect size estimation.<br>Bayesian linemodels calculation - https://github.com/mjpirinen/linemodels<br>PRS logistic regression was computed with LDLT-decomposition using the 'fastglm' R package (https://CRAN.R-project.org/package=fastglm). The association between the standardized PGS vector and each endpoint was adjusted for Sex, Age, Age2, the first six principal components, and genotyping array features.

For manuscripts utilizing custom algorithms or software that are central to the research but not yet described in published literature, software must be made available to editors and reviewers. We strongly encourage code deposition in a community repository (e.g. GitHub). See the Nature Portfolio guidelines for submitting code & software for further information.
